# The Treatment of Epilepsy

**Published:** 1889-12

**Authors:** 


*The Treatment of Epilepsy.* By WILLIAM ALEXANDER, M.D., F.R.C.S. Pp. 220. Edinburgh and London: YOUNG J. PENTLAND. 1889.

In 1882 Dr. Alexander introduced to the profession the operation of ligature of the vertebral arteries for the cure of epilepsy. This operation he has abandoned, as proving of "too temporary benefit to warrant its continuance." This work is written with the view of putting forward another operation with the same view; viz., removal of the cervical ganglia of the sympathetic.

The author has entered on his task fully equipped in theoretical reasoning and in practical manipulation. It is nearly always possible to find faults in the logic of a special pleader; but when the pleadings in the author's case lead to practical issues which promise to be beneficial, and in the critic's case leave us where we were, it would be ungracious to seek for such faults. The operation has been worked out with much care, and is fully and clearly described. The cases are admirably recorded and illustrated, and the results in the twenty-four cases described are decidedly favourable.

The chapter on the General Management of Epileptics is peculiarly interesting. The establishment of the Colony of Epileptics at Maghull, near Liverpool, is highly creditable to the author's mind and heart.

